# Limited prognostic value of the neutrophil-to-lymphocyte ratio compared with severity scores for mortality prediction in elderly critically ill patients

**DOI:** 10.62675/2965-2774.20260006

**Published:** 2026-09-17

**Authors:** Martín Angulo, Mauricio Ferreira, Andrés Rodríguez, Gastón Burghi

**Affiliations:** 1 Universidad de la República Facultad de Medicina Departamento de Fisiopatología Montevideo Uruguay Departamento de Fisiopatología, Hospital de Clínicas, Facultad de Medicina, Universidad de la República - Montevideo, Uruguay.; 2 Universidad de la República Hospital de Clínicas Laboratorio de Exploración Funcional Respiratoria Montevideo Uruguay Laboratorio de Exploración Funcional Respiratoria, Centro de Tratamiento Intensivo, Hospital de Clínicas, Facultad de Medicina, Universidad de la República - Montevideo, Uruguay.; 3 Centro de Asistencia Médica de Cerro Largo Unidad de Cuidados Intensivos Cerro Largo Uruguay Unidad de Cuidados Intensivos, Centro de Asistencia Médica de Cerro Largo - Cerro Largo, Uruguay.; 4 Universidad de la República Unidad Académica de Medicina Intensiva Hospital de Clínicas Montevideo Uruguay Unidad Académica de Medicina Intensiva, Hospital de Clínicas, Facultad de Medicina, Universidad de la República - Montevideo, Uruguay.

## INTRODUCTION

Predicting mortality in specific subgroups of critically ill patients remains challenging. Elderly patients represent a growing population in intensive care units (ICUs) and display distinctive characteristics, including increased vulnerability related to a high burden of comorbidities. However, elderly patients do not necessarily present higher mortality compared with other age groups.^(1)^

Severity-of-illness scores such as Simplified Acute Physiology Score II (SAPS II), Acute Physiology and Chronic Health Evaluation II (APACHE II), and Sequential Organ Failure Assessment (SOFA) have demonstrated adequate predictive performance for mortality in elderly ICU patients.^(2,3)^ In parallel, inflammation-related indices derived from the complete blood count (CBC) – particularly the neutrophil-to-lymphocyte ratio (NLR) – have been associated with mortality in several patient populations, including burned and oncological patients.^(4)^ Nevertheless, the prognostic value of NLR in elderly critically ill patients remains a matter of debate.

The aim of this study was to evaluate the performance of different severity scores, their combination, and the potential incremental value of adding the NLR to mortality prediction models in elderly ICU patients overall and in the subgroup with sepsis.

## METHODS

We conducted a retrospective, institutional review board-approved study at the *Centro de Asistencia Médica de Cerro Largo* (CAMCEL) Hospital (Melo, Uruguay). All patients aged ≥ 65 years admitted to the ICU between January 1, 2020, and December 31, 2023 were included. Patients with positive severe acute respiratory syndrome coronavirus 2 (SARS-CoV-2) tests or missing data required for score calculation were excluded. SAPS II, APACHE II, and SOFA scores were computed using the worst values within the first 24 hours of ICU admission. The NLR was derived from the admission CBC.

Statistical analyses were performed using IBM SPSS Statistics, version 24 (IBM Corp.), and GraphPad Prism, version 10 (GraphPad Software). Data are presented as median (25th-75th percentile) or absolute frequency (%). Group comparisons were performed using the Mann-Whitney U test or Chi-squared test, as appropriate. Prognostic performance was assessed using binary logistic regression and Receiver Operating Characteristic (ROC) curve analyses. Statistical significance was defined as p < 0.05. The same analyses were performed in the subgroup of patients admitted with sepsis, defined according to the Sepsis-3 consensus criteria.

## RESULTS

A total of 411 patients were included. Median age was 73 (69 - 79) years, and 159 patients (38.7%) died in the ICU. Baseline characteristics are shown in table 1. Non-survivors exhibited significantly higher SAPS II, APACHE II, and SOFA scores than survivors. The NLR values were also significantly higher in non-survivors (Figure 1A-C).

**Table 1:** Patient characteristics

Variable		Total N = 411	Survivors n = 252	Non-survivors n = 159	p value
Age (years)		73 (69 - 79)	73 (69 - 78)	74 (70 - 81)	0.08
Gender					0.3
	Female	174 (42.3)	102 (40.5)	72 (45.3)	
	Male	237 (57.7)	150 (59.5)	87 (54.7)	
Comorbidities					
	Hypertension	352 (85.6)	215 (85.3)	137 (86.2)	0.8
	History of smoking	149 (36.3)	103 (40.9)	46 (28.9)	0.01
	Diabetes mellitus	142 (34.5)	88 (34.9)	54 (34)	0.8
	Coronary artery disease	120 (29.2)	73 (29)	47 (29.6)	0.9
	Chronic obstructive pulmonary disease	66 (16.1)	39 (15.5)	27 (17)	0.7
	Malignancy	55 (13.4)	36 (14.3)	19 (11.9)	0.5
	Chronic kidney disease	50 (12.2)	32 (12.7)	18 (11.3)	0.7
Reason for ICU admission					< 0.0001
	Cardiovascular disease	141 (34.3)	93 (36.9)	48 (30.2)	
	Sepsis	124 (30.2)	58 (23)	66 (41.5)	
	Neurologic disease	54 (13.1)	32 (12.7)	22 (13.8)	
	Elective surgery	44 (10.7)	36 (14.3)	8 (5)	
	Respiratory disease	17 (4.1)	8 (3.2)	9 (5.7)	
	Trauma	7 (1.7)	5 (2)	2 (1.3)	
	Miscellaneous	24 (5.8)	20 (7.9)	4 (2.5)	
SAPS II		60 (37 - 78)	42 (32 - 62)	82 (71 - 92)	< 0.0001
APACHE II		26 (16 - 34)	18 (13 - 26)	35 (30 - 40)	< 0.0001
SOFA		10 (6 - 14)	6 (4 - 10)	14 (13 - 16)	< 0.0001
Neutrophil count (x10^9^/L)		8.9 (5.8 - 12.8)	8.6 (5.6 - 11.6)	9.9 (6.1 - 15.6)	0.01
Lymphocyte count (x10^9^/L)		1.1 (0.7 - 2.1)	1.2 (0.7 - 2.1)	1.0 (0.6 - 1.9)	0.03
Neutrophil-to-lymphocyte ratio		7.1 (3.8 - 15.6)	6.1 (3.3 - 14)	9.4 (4.5 - 17.4)	0.004
Renal replacement therapy		33 (8)	21 (8.3)	12 (7.5)	0.8
Vasopressor use		155 (37.7)	35 (13.9)	120 (75.5)	< 0.0001
Invasive mechanical ventilation		238 (57.9)	84 (33.3)	154 (96.9)	< 0.0001
Mechanical ventilation duration (days)		2 (1 - 7)	4 (2 - 10)	2 (1 - 5)	< 0.0001
ICU length of stay (days)		2 (1 - 6)	3 (1 - 6)	2 (1 - 6)	0.3
ICU mortality		159 (38.7)	0 (0)	159 (100)	N/A

ICU - intensive care unit; SAPS II - Simplified Acute Physiology Score II; APACHE II - Acute Physiology and Chronic Health Evaluation II; SOFA - Sequential Organ Failure. Data are presented as n (%) for categorical variables and median (25^th^ - 75^th^ percentile) for continuous variables. p value is shown for comparison between survivors and non-survivors (Mann Whitney U test or Chi-squared test).

**Figure 1:**
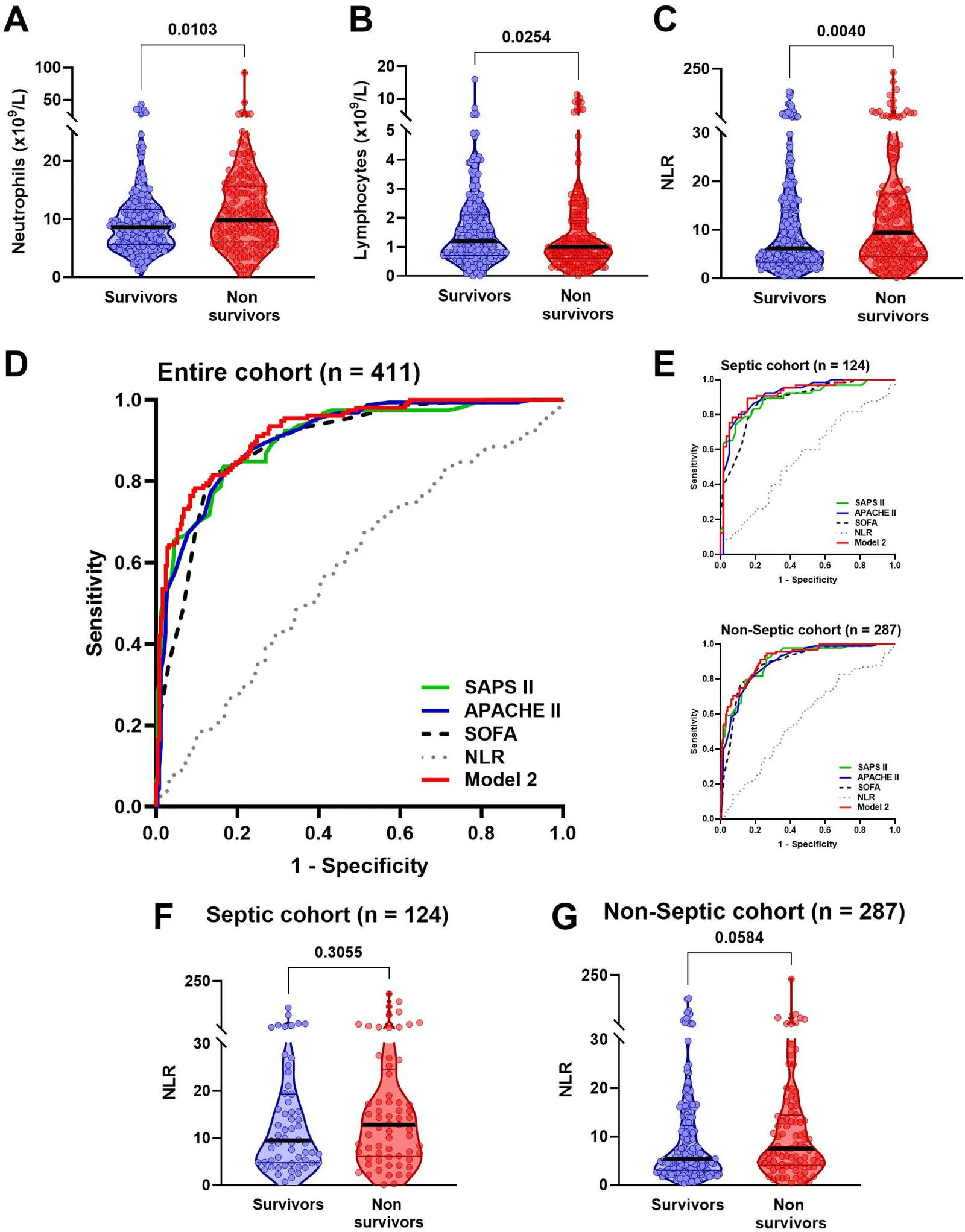
Inflammatory markers and prognostic score performance for intensive care unit mortality in the entire cohort and according to sepsis status.

NLR - neutrophil-to-lymphocyte ratio; SAPS II - Simplified Acute Physiology Score II; APACHE II - Acute Physiology and Chronic Health Evaluation II; SOFA - Sequential Organ Failure Assessment.

(A-C) Distribution of absolute neutrophil count, lymphocyte count, and neutrophil-to-lymphocyte ratio in intensive care unit survivors and non-survivors. (D) ROC curves comparing the discriminative performance of Simplified Acute Physiology Score II, Acute Physiology and Chronic Health Evaluation II, Sequential Organ Failure Assessment, neutrophil-to-lymphocyte ratio, and the combined model including Acute Physiology and Chronic Health Evaluation II, Simplified Acute Physiology Score II, Sequential Organ Failure Assessment, and neutrophil-to-lymphocyte ratio (Model 2) intensive care unit mortality in the entire cohort (n = 411). (E) ROC curves for the same models in septic (n = 124) and non-septic (n = 287) subgroups. (F-G) Distribution of neutrophil-to-lymphocyte ratio according to survival status in septic and non-septic patients, respectively. Violin plots display individual data points and median values. Group comparisons were performed using the Mann-Whitney U test.

Among individual scores, SAPS II, APACHE II, and SOFA demonstrated good discrimination for ICU mortality, with areas under the ROC curve (AUC) of 0.906, 0.905 and 0.892, respectively (p < 0.0001 for all). A combined model including SAPS II, APACHE II, and SOFA (Model 1) improved predictive performance (AUC 0.919, p < 0.0001) and showed good calibration.

Total neutrophil count, lymphocyte count and NLR exhibited poor discriminatory performance (AUC 0.575, p = 0.01; AUC 0.435, p = 0.03; and AUC 0.585, p = 0.004, respectively). Adding NLR to the combined severity score model (Model 2) resulted in only a modest incremental improvement (AUC 0.923, p < 0.0001), yielding the best overall statistical fit but with limited clinical relevance (Figure 1D).

In the subgroup of septic patients (n = 124), SAPS II, APACHE II, and SOFA also demonstrated good discrimination for ICU mortality, with AUCs of 0.894, 0.915, and 0.882, respectively (p < 0.0001 for all). The inclusion of NLR in this model (Model 2) did not significantly improve prognostic performance (AUC 0.921, p < 0.0001; Figure 1E-G).

## DISCUSSION

In contrast to some previous reports with case mix differences, our study demonstrates excellent predictive performance of SAPS II, APACHE II, SOFA, and their combination in a cohort of 411 elderly ICU patients.^(5)^ In comparison, NLR showed poor prognostic performance, and its addition to established severity scores did not meaningfully enhance mortality prediction in this population. These findings were consistent even in septic patients, a subgroup characterized by pronounced inflammatory activation. From a clinical perspective, although the inclusion of NLR produced a small statistical increase in model discrimination, the magnitude of this change was minimal and unlikely to translate into meaningful clinical benefit.

The limited predictive value of inflammation-based biomarkers in elderly patients may be explained by age-related immune alterations collectively referred to as immunosenescence. Immunosenescence is characterized by impaired neutrophil phagocytic and chemotactic capacity, as well as reduced lymphocyte migration, adhesion, and adaptive immune responsiveness. In parallel, elderly individuals often exhibit a state of chronic low-grade systemic inflammation ("inflammaging"), driven by increased oxidative stress, persistent DNA damage, and stem cell aging. This coexistence of immune dysfunction and chronic inflammation highlights the complexity of immune regulation in aging and may attenuate the prognostic signal of inflammation-derived markers such as NLR.^(6-9)^ Moreover, in the present study NLR was assessed only at ICU admission, which may not fully capture the dynamic inflammatory changes occurring during critical illness. Serial measurements or alternative inflammatory indices (e.g., platelet-to-lymphocyte ratio or systemic immune-inflammation index [SII]), as well as combinations of biomarkers, might provide additional prognostic information and deserve further investigation.

This study has several limitations. Its single-center design limits external generalizability. Additionally, the absence of frailty assessment – an important determinant of outcomes in elderly critically ill patients – may have influenced the observed associations.^(10)^ Frailty reflects diminished physiological reserve and may interact with both illness severity scores and inflammatory markers, potentially modifying their prognostic performance in this population.

## ETHICAL CONSIDERATIONS

The study was conducted in accordance with the ethical principles of the Declaration of Helsinki and was approved by the Ethics Committee of *Centro de Asistencia Médica de Cerro Largo*. The need for informed consent was waived by the local Institutional Review Board due to the retrospective design of the study.

## Data Availability

The datasets used during the current study are available from the corresponding author on reasonable request.
